# Dysregulation of Circadian Markers, HAT1 and Associated Epigenetic Proteins, and the Anti-Aging Protein KLOTHO in Placenta of Pregnant Women with Chronic Venous Disease

**DOI:** 10.3390/jpm15030107

**Published:** 2025-03-09

**Authors:** Oscar Fraile-Martinez, Cielo García-Montero, Tatiana Pekarek, Julia Bujan, Silvestra Barrena-Blázquez, Eva Manuela Pena-Burgos, Laura López-González, Leonel Pekarek, Raul Díaz-Pedrero, Juan A. De León-Luis, Coral Bravo, Melchor Álvarez-Mon, Miguel A. Saez, Natalio García-Honduvilla, Miguel A. Ortega

**Affiliations:** 1 Department of Medicine and Medical Specialities, Faculty of Medicine and Health Sciences, University of Alcalá, Networking Research Center on for Liver and Digestive Diseases (CIBEREHD), 28801 Alcala de Henares, Spain; oscarfra.7@gmail.com (O.F.-M.); cielo.gmontero@gmail.com (C.G.-M.); tatianapekarek@gmail.com (T.P.); mjulia.bujan@uah.es (J.B.); leonel.pekarek@gmail.com (L.P.); mademons@gmail.com (M.Á.-M.); msaega1@oc.mde.es (M.A.S.); natalio.garcia@uah.es (N.G.-H.); 2 Ramón y Cajal Institute of Sanitary Research (IRYCIS), 28034 Madrid, Spain; silvebarrena@gmail.com (S.B.-B.); laura.lgonzalez@uah.es (L.L.-G.); raul.diazp@uah.es (R.D.-P.); 3 Department of Nursing and Physiotherapy, Faculty of Medicine and Health Sciences, University of Alcalá, 28801 Alcala de Henares, Spain; 4 Pathological Anatomy Service, University Hospital Gregorio Marañón, 28009 Madrid, Spain; evamanuela.pena@salud.madrid.org; 5 Department of Surgery, Medical and Social Sciences, Faculty of Medicine and Health Sciences, University of Alcalá, 28801 Alcala de Henares, Spain; 6 Department of Public and Maternal and Child Health, School of Medicine, Complutense University of Madrid, 28040 Madrid, Spain; jaleon@ucm.es (J.A.D.L.-L.); cbravoarribas@gmail.com (C.B.); 7 Department of Obstetrics and Gynecology, University Hospital Gregorio Marañón, 28009 Madrid, Spain; 8 Health Research Institute Gregorio Marañón, 28009 Madrid, Spain; 9 Immune System Diseases-Rheumatology and Internal Medicine Service, University Hospital Prince of Asturias, Networking Research Center on for Liver and Digestive Diseases (CIBEREHD), 28806 Alcala de Henares, Spain; 10 Pathological Anatomy Service, University Hospital Gómez-Ulla, 28806 Alcala de Henares, Spain

**Keywords:** chronic venous disease (CVD), placenta, pregnancy, histopathology, circadian alterations, epigenetic changes, aging

## Abstract

**Background:** Chronic venous disease (CVD) is a vascular disorder common among pregnant women, due to the impairment in the venous function associated with the mechanical, hemodynamical, and hormonal changes that occur during pregnancy. CVD is linked to venous hypertension, inflammation, oxidative stress, and hypoxia, which alter placental structure and function, as demonstrated in previous works. The placenta fulfills several roles in fetal development and maternal well-being by mediating nutrient exchange; acting as a mechanical, chemical, and immunological shield; and producing essential hormones, making it crucial to investigate the effects of CVD in this organ. **Patients and methods:** This work specifically analyzes the gene expression of circadian markers (*CLOCK*, *BMAL1*, *PER1*, and *PER2*), epigenetic regulators (*HAT1* and associated molecules like histones *H3*, *H4*, *RBBP7,* and *ASF1*), and the anti-aging protein *KLOTHO* in placental tissue of pregnant women with CVD (CVD-PW, N = 98) compared to healthy pregnant controls (HC-PW, N = 82), using RT-qPCR and immunohistochemistry (IHC) to determine protein expression. **Results:** Our study demonstrates that the placentas of CVD-PW exhibit the reduced gene and protein levels of circadian regulators (clock, bmal1, per1, and per2), increased expression of hat1 and related proteins (h3, h4, rbbp7, and asf1), and decreased klotho expression, indicative of accelerated aging. **Conclusions:** These findings highlight profound molecular disturbances in the placentas of women with CVD, offering insights into the disease’s pathophysiology and potential implications for maternofetal well-being. While this study deepens our understanding of the relationship between CVD and placental dysfunction, further research is required to fully elucidate these mechanisms and their long-term effects.

## 1. Introduction

Chronic venous disease (CVD) is a prevalent vascular disorder characterized by venous hypertension and structural vein abnormalities, such as telangiectasias, reticular veins, and varicose veins (VVs), commonly affecting the lower extremities [1,2,3]. Pregnancy is considered a major risk factor for developing CVD, as the hormonal, mechanic, and hemodynamic changes that occur during this period contribute to the development of this condition [4,5,6]. Indeed, according to epidemiological data, CVD affects between 15 and 50% of pregnant women, with some important factors, such as age, multiparity, and family history of CVD, influencing this variation [4,7,8]. After pregnancy, approximately 30% of women remain with this condition, evidencing the impact of pregnancy in the venous system [8].

CVD during pregnancy significantly impairs both the venous system and systemic health, as increased venous pressure and hormonal changes lead to venous dilation, impaired blood flow, and swelling in the lower limbs [4,9]. These alterations in venous hemodynamics are exacerbated by the growing uterus, which compresses the inferior vena cava and iliac veins, further impeding venous return [10]. Systemically, these alterations contribute to inflammation, oxidative stress, and endothelial dysfunction, which can disrupt normal vascular function, impairing maternal and fetal well-being [11,12,13,14]. Past works have evidenced that CVD during pregnancy promotes significant changes in the cytoarchitecture and function of the placental tissue and other maternofetal structures, such as the umbilical cord [9,15]. The placenta is a pivotal organ during pregnancy, playing multiple roles during this period by facilitating the exchange of nutrients, oxygen, and waste products between the mother and fetus, ensuring proper fetal development. It also functions as an endocrine organ, producing hormones that regulate pregnancy, support fetal growth, and influence fetal and maternal health during and after this period [9,16,17]. Therefore, based on the potential alterations that occur in this organ during pregnancy, it is critical to better understand the impact and consequences of CVD.

Proper placental function depends on tightly regulated molecular pathways, including circadian and epigenetic mechanisms. Circadian regulation, driven by core clock genes such as Circadian Locomotor Output Cycles Kaput (*CLOCK*), Brain and Muscle ARNT-Like 1, (*BMAL1*)—also known as *ARNTL*— period 1 (*PER1*), and *PER2*, is essential for synchronizing placental functions with maternal and fetal needs [18,19]. Similarly, epigenetic processes are crucial for the dynamic gene expression necessary for placental development and physiology [20,21]. Histone acetyl transferase 1 (HAT1) is responsible for catalyzing the acetylation of histone 4 (H4) in a recently assembled H3/H4 dimer in the cytoplasm, thus playing a critical role in epigenetic regulation [22,23]. In their epigenetic roles, HAT1 and H3/H4 interact with other critical proteins, such as the Anti-Silencing Function 1 (ASF1) and the retinoblastoma-Binding Protein 7 (RBBP7). Alterations in circadian and epigenetic function in the placental tissue have been implicated in various obstetric complications [24,25,26], suggesting the potential of exploring these processes in women who develop CVD during pregnancy.

On the other hand, aging is an inevitable physiological process that occurs in all tissues, including the placenta, which has a strictly limited lifespan of 40 weeks. While placental aging is a natural part of pregnancy necessary for labor induction, its premature or delayed occurrence is linked to complications, such as preeclampsia, preterm birth, stillbirth, and other placental dysfunctions, highlighting the importance of understanding its molecular mechanisms in relation to both pregnancy outcomes and long-term health [27]. In this sense, alterations in the anti-aging protein KLOTHO within the placenta may indicate accelerated aging, further exacerbating placental dysfunction as demonstrated in previous works [28,29]. To the best of our knowledge, there are no studies specifically exploring possible disturbances in circadian rhythms, epigenetic regulation, and aging in the placental tissue of women who develop and suffer from CVD during pregnancy. Understanding how these mechanisms are disrupted in women with CVD during pregnancy could provide key insights into disease progression and potential therapeutic targets.

In this context, the purpose of the current study is to analyze gene and protein expression by performing real-time quantitative PCR (RT-qPCR) and immunohistochemistry (IHC), respectively, on circadian markers (clock, bmal1, per1, per2), epigenetic regulators (Hat1 and related molecules), and the anti-aging protein klotho in the placentas of pregnant women with CVD (CVD-PW), comparing them with healthy control pregnant women (HC-PW).

## 2. Patients and Methods

### 2.1. Study Design

A prospective, analytical, and observational study was carried out with 180 pregnant women in their third trimester. Of them, 82 participants comprised the HC-PW group, whereas 98 were diagnosed with CVD (CVD-PW) by using the clinical–etiological–anatomical–pathophysiological (CEAP) classification system [30]. The study adhered strictly to key ethical principles, including autonomy, beneficence, non-maleficence, and distributive justice. It was carried out in compliance with Good Clinical Practice guidelines and the ethical standards outlined in the latest Helsinki Declaration (2013) and the Oviedo Convention (1997). All participants received detailed information about the study and voluntarily provided written informed consent prior to their inclusion. The study protocol was approved by the Clinical Research Ethics Committee of the Central University Hospital of Defense, University of Alcalá (37/17).

### 2.2. Participants Enrolled

This study included women over the age of 18 who presented with third-trimester clinical manifestations of venous disease in the lower extremities, meeting the inclusion criteria of the CEAP classification at stage ≥ 1. Participants’ medical records were reviewed in detail, and their physical condition was assessed during a third-trimester consultation. Additionally, lower-limb ultrasound examinations were performed using a 7.5 MHz probe on a portable Eco-Doppler device (M-Turbo Eco-Doppler; SonoSite, Inc., Bothell, WA, USA) to evaluate venous function.

**Figure 1:**
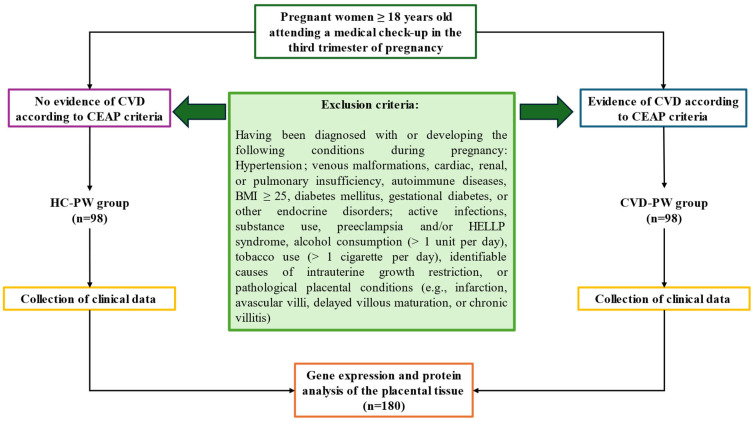
A diagram of the methods used for the the recruitment and stratification of groups (CVD-PW = pregnant women with chronic venous disease; HC-PW = healthy pregnant women without chronic venous disease).

Exclusion criteria encompassed individuals with prior diagnoses of hypertension, venous malformations, cardiac, renal, or pulmonary insufficiency, autoimmune diseases, a body mass index (BMI) ≥ 25, diabetes mellitus, gestational diabetes, or other endocrine disorders. Additionally, individuals with active infections, substance use (e.g., cannabis, heroin, cocaine, or amphetamines), preeclampsia and/or HELLP syndrome, alcohol consumption exceeding one unit per day, tobacco use of more than one cigarette per day, identifiable causes of intrauterine growth restriction, or pathological placental conditions (e.g., infarction, avascular villi, delayed villous maturation, or chronic villitis) were excluded. Cases that developed any exclusion criteria during the study period were also omitted. A diagram summarizing the methods used for recruitment and group stratification is presented in Figure 1.

The HC-PW group had a median maternal age of 34 years (IQR: 20–41 years) and a median gestational duration of 41 weeks (IQR: 39–42 weeks). In contrast, the CVD-PW group had a median maternal age of 33 years (IQR: 19–41 years) and a median gestational duration of 40 weeks (IQR: 39–41.5 weeks). There were no statistically significant differences between the groups in terms of prior pregnancies (HC: 37.8%; CVD: 53.1%), gestational age, delivery mode, abortion history, menstrual cycle regularity, or occupational status (as detailed in Table 1).

### 2.3. Sample Collection and Processing

Placental biopsies were collected from 180 patients at the time of delivery. Each placenta was divided into five fragments and processed using a scalpel to include a variety of cotyledons for each one. Two sterile tubes were used for sample storage: one containing RNAlater^®^ solution (Ambion; Thermo Fisher Scientific, Waltham, MA, USA) and the other containing Minimum Essential Medium (MEM; Thermo Fisher Scientific, Waltham, MA, USA) supplemented with 1% antibiotic/antimycotic solution (Streptomycin, Amphotericin B, and Penicillin; Thermo Fisher Scientific, Waltham, MA, USA). All procedures were carried out aseptically within a class II laminar flow hood (Telstar AV 30/70 Müller 220 V 50 MHz; Telstar; Azbil Corporation, Chiyoda-ku, Tokyo, Japan). Samples designated for gene expression analysis were submerged in 1 mL of RNAlater^®^ solution and stored at −80 °C, while those stored in MEM were reserved for histological and immunohistochemical studies.

To remove erythrocytes, samples were rehydrated with MEM without antibiotics. Subsequently, the samples were cut into 2 cm sections and fixed in F13 solution (60% ethanol, 20% methanol, 7% polyethylene glycol, and 13% distilled water) following standardized protocols [12]. After fixation, the samples were placed in molds and embedded in paraffin. Once the paraffin had solidified, 5 μm thick sections were cut using an HM 350 S rotary microtome (Thermo Fisher Scientific, Waltham, MA, USA). The sections were then floated in a heated water bath and mounted onto glass slides pre-treated with 10% poly-L-lysine to enhance adhesion.

### 2.4. Gene Expression Analysis

RNA was extracted using the guanidinium thiocyanate–phenol–chloroform method to analyze mRNA expression levels of selected target genes [12]. Complementary DNA (cDNA) was synthesized through reverse transcription (RT) from RNA samples standardized to a concentration of 50 ng/µL. Each sample was placed in an oligo-dT solution and denatured at 65 °C for 10 min. A RT mix—including first-strand buffer, deoxyribonucleotide triphosphates, dithiothreitol, DNase- and RNase-free water, an RNase inhibitor, and reverse transcriptase—was then mixed.

The RT process was conducted using a G-Storm GS1 thermal cycler. Samples were first incubated at 70 °C for 15 min to inactivate the reverse transcriptase enzyme, gradually cooled to 4 °C, and subsequently subjected to cDNA synthesis at 37 °C for 75 min. To ensure the LACK of genomic DNA contamination, a negative control RT reaction was performed by replacing the RT enzyme with DNase- and RNase-free water. The resulting cDNA was diluted 1:20 in DNase- and RNase-free water and stored at −20 °C.

Specific primers for the genes of interest were designed using Primer-BLAST (v. 0.4.0) and AutoDimer (v. 1.0) following standardized protocols [31,32]. The TATA-box-binding protein (*TBP*) gene was used as the normalization control [33]. Relative mRNA expression was quantified via RT-qPCR on a StepOnePlus™ System (Applied Biosystems; Thermo Fisher Scientific, Inc.) using the relative standard curve method. Each reaction, performed in a MicroAmp^®^ 96-well plate, included iQ™ SYBR^®^ Green Supermix (Bio-Rad Laboratories, Inc., Hercules, CA, USA), forward and reverse primers, and RNase- and DNase-free water. The thermocycling conditions consisted of multiple denaturation, annealing, and elongation cycles, with a dissociation curve generated for additional analysis.

Fluorescence data were recorded during the dissociation curve and at the end of each amplification cycle. A standard curve was constructed using serially diluted samples to quantify relative expression levels, including TBP expression on every plate. RT-qPCR was conducted twice for each placental tissue sample. For each sample, the Ct values of the gene of interest and the housekeeping gene (*TBP*) were determined, and the ΔCt was calculated by subtracting the Ct of the housekeeping gene from the Ct of the gene of interest. The relative expression was then calculated using the formula 2^(−ΔCt) and expressed as a percentage, where the expression of the gene of interest was compared to the housekeeping gene. The results were visualized using boxplots, representing the distribution of relative expression percentages in both the CVD and HC pregnant women, following established methodologies [15]. In Table 2, genes of interests and their primers are represented.

### 2.5. Protein Expression Analysis and Histopathological Evaluation

In accordance with established protocols [15], the avidin–biotin complex technique was performed to detect antigen–antibody reactions in paraffin-embedded placental specimens. Specific antibody details are outlined in Table 3 of the study protocol. Placental sections were incubated with the primary antibody for 90 min, followed by overnight treatment at 4 °C with 3% BSA Blocker in PBS to reduce nonspecific binding. Subsequently, samples were incubated for 90 min at room temperature with a biotin-conjugated secondary antibody diluted in PBS (Table 2). ExtrAvidin^®^-Peroxidase (Sigma-Aldrich, St. Louis, MO, USA), diluted 1:200 in PBS, was applied for 60 min at room temperature.

To visualize protein expression levels, a chromogenic diaminobenzidine (DAB) substrate kit was freshly prepared and used. The kit consisted of 5 mL distilled water, four drops of DAB, two drops of buffer, and two drops of hydrogen peroxide, producing a brown stain for signal detection. Negative control sections for proteins of interest underwent the same protocol with the primary antibody replaced by a PBS blocking solution. For 15 min, Carazzi hematoxylin was used to provide contrast in histological samples.

Placental specimens were analyzed under a Zeiss Axiophot optical microscope (Zeiss GmbH, Jena, Germany), examining five tissue sections and ten randomly selected fields of view per patient. Protein expression was scored using the immunoreactive score (IRS) [34], defining positive staining as occupying at least 5% of the sample. Two independent histologists performed a double-blinded evaluation and assigned scores: 0 to 1 for minimal staining (≤25%), 2 to 4 for moderate staining (25–65%), and 3 to 4 for intense staining (65–100%). Discrepancies were resolved by the intervention of a third pathologist.

### 2.6. Statistical Analysis

Data analysis was performed using GraphPad Prism^®^ v6.0 software (GraphPad, Inc., San Diego, CA, USA). The normality of data distributions was evaluated using the Kolmogorov–Smirnoff test (all *p* < 0.001). Due to significant deviations from normality, non-parametric tests were applied, and results were presented as medians with interquartile ranges. Group comparisons were made using the Mann–Whitney U test. Statistical significance was determined with *p*-values of *p* < 0.05, *p* < 0.01, and *p* < 0.001.

## 3. Results

### 3.1. Placentas of Women Who Undergo Chronic Venous Disease During Pregnancy Display Decreased Expression of Key Circadian Markers

**Figure 2:**
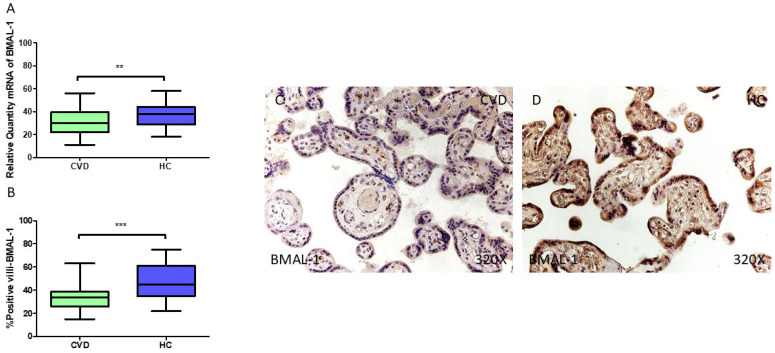
(**A**) Bmal1 mRNA expression levels in pregnant females who were diagnosed with CVD compared to HC group (** *p* < 0.01). (**B**) IRS scores assessing Bmal1 expression in placental tissue of women with CVD and without this condition (*** *p* < 0.0001). (**C**,**D**) Representative immunostaining images displaying Bmal1 expression in placental villi from CVD and HC samples.

Our findings indicate a significant reduction in *BMAL1* gene expression (RT-qPCR) in the placental tissue of pregnant women with CVD (** *p* = 0.0012; CVD = 30.034 [11.065–56.031], HC = 38.065 [18.065–58.062], Figure 2A). The histological analysis of the placental villi showed a marked decrease in Bmal1 protein expression in women with CVD (*** *p* < 0.0001; CVD = 34.000 [15.000–63.000], HC = 45.000 [22.000–75.000], Figure 2B). Bmal1 expression was more pronounced in the placental villi of the HC women, particularly within the syncytiotrophoblast layer, compared to those with CVD. In CVD cases, Bmal1 protein expression was observed only in certain cells within the inner part of the placental villi (Figure 2C,D).

**Figure 3:**
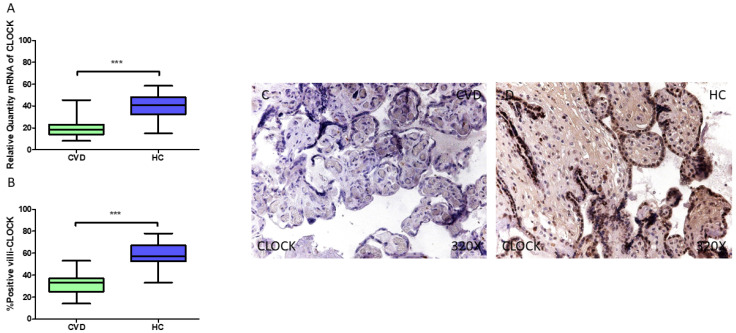
(**A**) CLOCK mRNA expression levels in pregnant females who were diagnosed with CVD compared to HC group (*** *p* < 0.0001). (**B**) IRS scores assessing CLOCK expression in placental tissue of women with CVD and without this condition (*** *p* < 0.0001). (**C**,**D**) Representative immunostaining images displaying CLOCK expression in placental villi from CVD and HC samples.

In a similar manner, the gene expression analysis detected a statistically significant downregulation in *CLOCK* (RT-qPCR) within the placental tissue of pregnant women who develop CVD during pregnancy (*** *p* < 0.0001; CVD = 18.094 [8.156–45.454], HC = 41.022 [15.032–58.456], Figure 3A). The immunohistochemical analysis of placental villi revealed a significant reduction in clock protein expression (%) in the villi of women with CVD (*** *p* < 0.0001; CVD = 33.000 [14.000–53.000], HC = 57.000 [33.000–78.000], Figure 3B). Clock tissue expression was notably increased across all placental villi in the HC group compared to the CVD group (Figure 3C,D).

**Figure 4:**
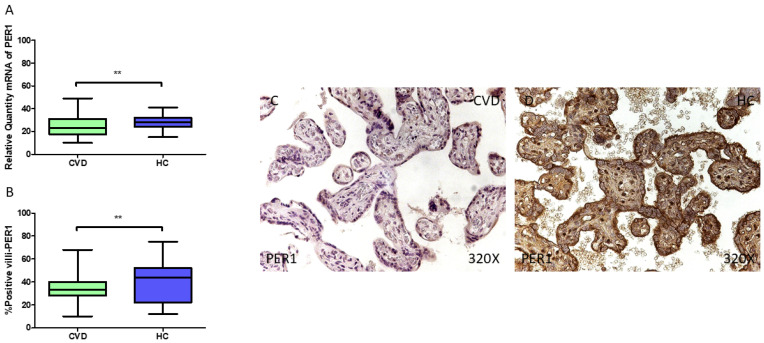
(**A**) PER1 mRNA expression levels in pregnant females who were diagnosed with CVD compared to HC group (** *p* < 0.01). (**B**) IRS scores assessing PER1 expression in placental tissue of women with CVD and without this condition (** *p* < 0.01). (**C**,**D**) Representative immunostaining images displaying PER1 expression in placental villi from CVD and HC samples.

Furthermore, our results demonstrate a statistically significant decrease in *PER1* gene expression (RT-qPCR) in the placental tissue of pregnant women with CVD (** *p* = 0.002; CVD = 23.022 [10.062–49.065], HC = 28.065 [15.017–41.079], Figure 4A). The histological examination of the placental villi revealed a notable reduction in Per1 protein expression (%) in women with CVD (** *p* = 0.009; CVD = 33.000 [10.000–68.000], HC = 44.000 [12.000–75.000], Figure 4B). The tissue expression of Per1 was significantly higher in all placental villi of the HC women compared to the CVD group (Figure 4C,D).

**Figure 5:**
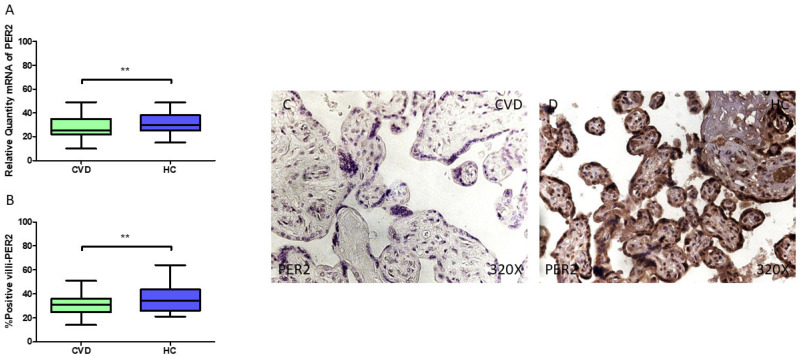
(**A**) PER2 mRNA expression levels in pregnant females who were diagnosed with CVD compared to HC group (** *p* < 0.01). (**B**) IRS scores assessing PER2 expression in placental tissue of women with CVD and without this condition (** *p* < 0.01). (**C**,**D**) Representative immunostaining images displaying Bmal1 expression in placental villi from CVD and HC samples.

Lastly, our results demonstrate a statistically significant reduction in *PER2* gene expression (RT-qPCR) in the placental tissue of pregnant women with CVD (** *p* = 0.0018; CVD = 25.408 [10.052–49.061], HC = 30.070 [15.062–48.878], Figure 5A). The histological analysis of the placental villi revealed a significant downregulation in Per2 protein expression (%) in the chorionic villi of women with CVD (** *p* = 0.0019; CVD = 31.000 [14.000–51.000], HC = 34.500 [21.000–64.000], Figure 5B). Per2 tissue expression was notably higher in all the placental villi of the HC women compared to the CVD group (Figure 5C,D).

### 3.2. The Placentas of Women with Chronic Venous Disease During Pregnancy Show Evidence of Altered Epigenetic Markers

**Figure 6:**
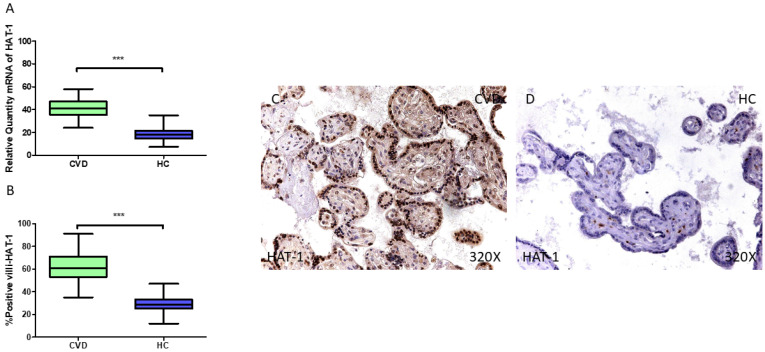
(**A**) HAT1 mRNA expression levels in pregnant females who were diagnosed with CVD compared to HC group (*** *p* < 0.0001). (**B**) IRS scores assessing HAT1 expression in placental tissue of women with CVD and without this condition (*** *p* < 0.0001). (**C**,**D**) Representative immunostaining images displaying HAT1 expression in placental villi from CVD and HC samples.

In parallel, our findings do support a significant rise in *HAT1* gene expression in the placentas of the CVD-PW (*** *p* < 0.001; CVD = 41.036 [24.013–58.062], HC = 18.090 [7.300–35.055], Figure 6A). The histological analysis of the placental villi revealed a significant rise in Hat1 protein expression (%) in the chorionic villi of women with CVD (*** *p* < 0.001; CVD = 61.000 [35.000–91.000], HC = 28.500 [12.000–47.000], Figure 6B). Hat1 tissue expression was notably increased in all the placental villi of women affected by CVD compared to the HC, especially within the syncytiotrophoblast layer (Figure 6C,D).

**Figure 7:**
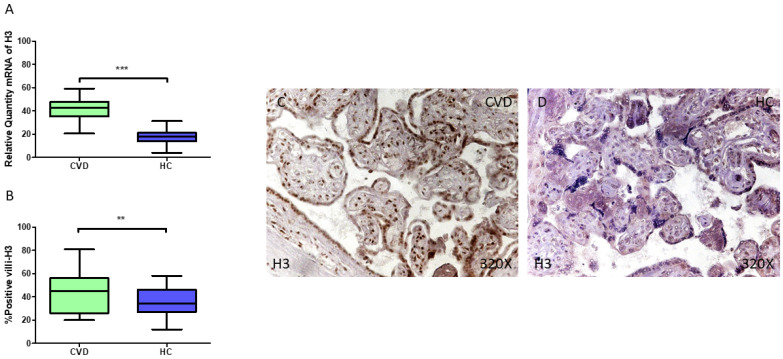
(**A**) H3 mRNA expression levels in pregnant females who were diagnosed with CVD compared to HC group (*** *p* < 0.0001). (**B**) IRS scores assessing H3 expression in placental tissue of women with CVD and without this condition (** *p* < 0.01). (**C**,**D**) Representative immunostaining images displaying H3 expression in placental villi from CVD and HC samples.

At the same time, our findings show a statistically significant increase in *H3* gene expression (RT-qPCR) in the placental tissue of pregnant women with CVD (*** *p* < 0.001; CVD = 42.535 [20.515–59.020], HC = 18.065 [4.065–31.202], Figure 7A). The histological analysis of placental villi revealed a marked increase in H3 protein expression (%) in the chorionic villi of women with CVD (** *p* = 0.0054; CVD = 45.000 [20.000–81.000], HC = 34.500 [12.000–58.000], Figure 7B). H3 tissue expression was significantly higher in all the placental villi of the women affected by CVD (Figure 7C,D).

**Figure 8:**
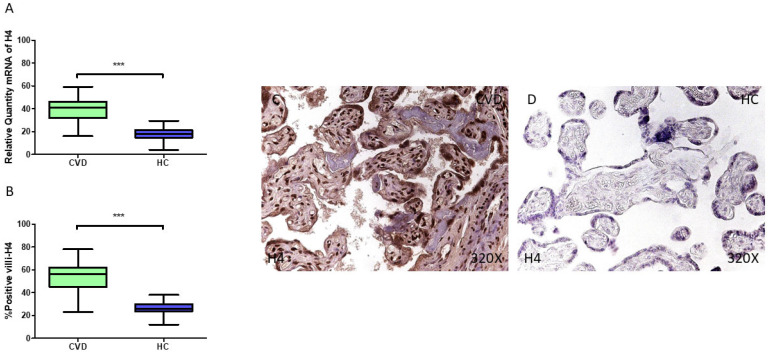
(**A**) H4 mRNA expression levels in pregnant females who were diagnosed with CVD compared to HC group (*** *p* < 0.0001). (**B**) IRS scores assessing H4 expression in placental tissue from women with CVD and without this condition (*** *p* < 0.0001). (**C**,**D**) Representative immunostaining images displaying H4 expression in placental villi from CVD and HC samples.

Our gene analysis reports upregulated *H4* expression (RT-qPCR) in the placentas of women diagnosed with CVD during pregnancy (*** *p* < 0.001; CVD = 41.062 [16.065–59.068], HC = 17.777 [4.051–29.479], Figure 8A). The histopathological analysis of placental villi displays a significant augmentation in H4 protein expression (%) in women with CVD (*** *p* < 0.001; CVD = 56.000 [23.000–78.000], HC = 26.000 [12.000–38.000], Figure 8B). The microscopic examination of H4 reveals that this protein was markedly overexpressed in the chorionic villi of women with CVD compared to HC group, with a strong detection in the syncytiotrophoblast layer (Figure 8C,D).

**Figure 9:**
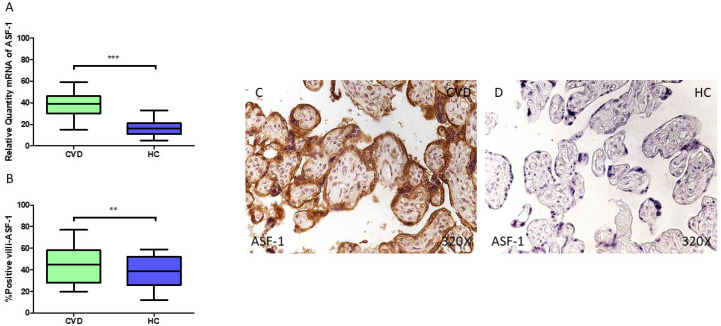
(**A**) ASF-1 mRNA expression levels in pregnant females who were diagnosed with CVD compared to HC group (*** *p* < 0.0001). (**B**) IRS scores assessing ASF-1 expression in placental tissue of women with CVD and without this condition (** *p* < 0.01). (**C**,**D**) Representative immunostaining images displaying ASF-1 expression in placental villi from CVD and HC samples.

Moreover, our findings demonstrate an elevation in *ASF1* gene expression (RT-qPCR) which is statistically significant within the placental tissue of pregnant women diagnosed with CVD (** *p* < 0.001; CVD = 39.073 [15.032–59.065], HC = 16.078 [5.062–33.068], Figure 9A). The microscopic evaluation of the villi showed a substantial increase in ASF1 protein levels (%) in the chorionic villi of CVD-affected women (** *p* = 0.0041; CVD = 45.000 [20.000–77.000], HC = 39.000 [12.000–59.000], Figure 9B). The distribution of the ASF1 protein was significantly more pronounced across all placental villi in the CVD cases compared to the HC cases, with a particularly strong presence in the syncytiotrophoblast layer (Figure 9C,D).

**Figure 10:**
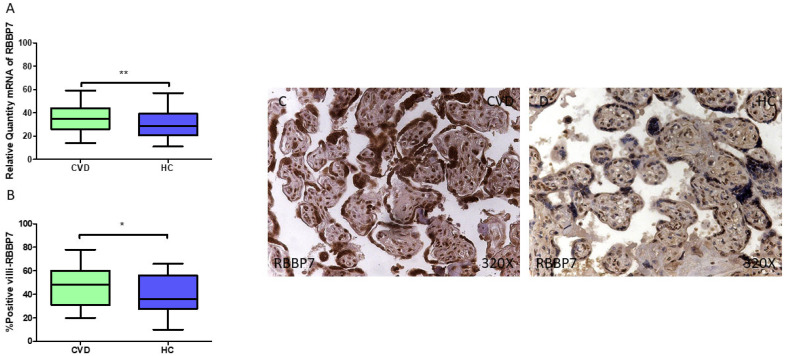
(**A**) RBBP7 mRNA expression levels in women with CVD compared to HC group (** *p* < 0.01). (**B**) IRS scores assessing RBBP7 expression in placental tissue of women with CVD and without this condition (* *p* < 0.05). (**C**,**D**) Representative immunostaining images displaying RBBP7 expression in placental villi from CVD and HC samples.

Finally, we observed a notable upregulation in *RBBP7* gene expression (RT-qPCR) in the placental tissue of pregnant women with CVD (** *p* = 0.0035; CVD = 35.032 [14.065–59.066], HC = 28.827 [11.007–57.006], Figure 10A). The histopathological analysis of the placental villi indicated a significant rise in RBBP7 protein expression (%) within the chorionic villi of women affected by CVD (* *p* = 0.0253; CVD = 48.000 [20.000–78.000], HC = 36.000 [10.000–66.000], Figure 10B). RBBP7 protein levels were markedly higher in all the placental villi of women with CVD, especially in the syncytiotrophoblasts, with a comparable distribution also noted in the inner regions of the placental villi (Figure 10C,D).

### 3.3. The Placentas of Women with Chronic Venous Disease During Pregnancy Exhibit Reduced Expression of the Anti-Aging Protein KLOTHO

**Figure 11:**
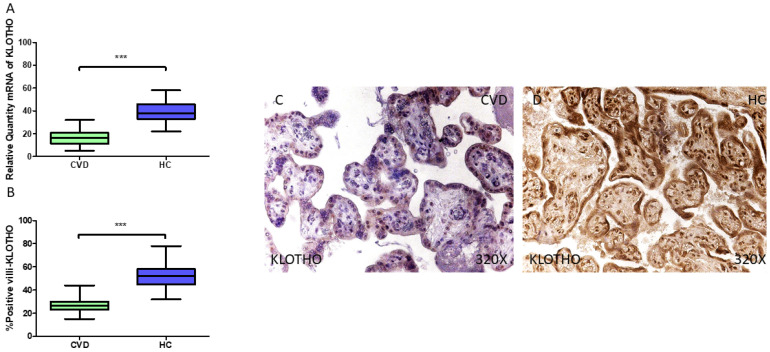
(**A**) KLOTHO mRNA expression levels in pregnant females who were diagnosed with CVD compared to HC group (*** *p* < 0.0001). (**B**) IRS scores assessing KLOTHO expression in placental tissue of women with CVD and without this condition (*** *p* < 0.0001). (**C**,**D**) Representative immunostaining images displaying KLOTHO expression in placental villi from CVD and HC samples.

Our findings indicate a statistically significant decline in *KLOTHO* gene expression (RT-qPCR) in the placental tissue of women diagnosed with CVD during pregnancy (** *p* < 0.001; CVD = 16.189 [5.061–32.013], HC = 38.063 [22.012–58.151], Figure 11A). The histological examination of the placental villi demonstrated a substantial reduction in KLOTHO protein levels (%) within the placental villi of women with CVD (*** *p* < 0.001; CVD = 26.500 [15.000–44.000], HC = 52.000 [32.000–78.000], Figure 11B). The KLOTHO protein was significantly more abundant throughout all the placental villi in the HC cases, whereas in CVD-affected women, its expression was minimal (Figure 11C,D).

## 4. Discussion

In the present work, we have demonstrated that the placentas of CVD-PW show an aberrant expression of circadian markers (clock, bmal1, per1, and per2), epigenetic regulators (HAT-1, H3, H4, rbbp7, and asf1), and the anti-aging protein klotho when compared to HC-PW. These results aid to gain further insights into the pathogenic consequences of CVD during pregnancy in the placental tissue of affected women.

This study suggests the possible implications of aberrant circadian regulation in the placental tissue of pregnant women experiencing CVD. Circadian rhythms are orchestrated by a “central clock” located in the suprachiasmatic nucleus (SCN) of the hypothalamus in response to photic (light–dark cycles) and non-photic stimuli [35]. However, circadian rhythms are endogenously generated in each organ of the body (peripheral clocks), including in the human placenta [36]. Collectively, the central and peripheral clocks show a dynamic, bidirectional, and complex interplay. During pregnancy, these interactions ultimately impact the health of the mother and offspring [37]. From a molecular perspective, various proteins, such as CLOCK, Bmal1, PER1, PER2, CRY1, CRY2, Rev-erbα, and casein kinase 1 epsilon (CK1ε), are responsible for the modulation of the circadian clock. Intracellular clock mechanisms rely on interacting positive and negative feedback loops, consisting of a core oscillator and accessory components. In the primary loop, the CLOCK-BMAL1 heterodimer activates the transcription of Per and Cry genes by binding to E-box regions in their promoters during the light phase [38,39]. Later, PER, and CRY proteins accumulate, form cytoplasmic complexes, and peak at the end of the light phase. These complexes move into the nucleus, where they inhibit CLOCK-BMAL1 activity, reducing Per and Cry transcription. As PER and CRY levels decline, their inhibition is lifted, and the cycle restarts. Therefore, the study of CLOCK, Bmal1, PER1, and PER2 as critical components of the circadian clock in the placental tissue of pregnant women who develop CVD represents a promising area of research in order to understand the effects of this condition during this period.

Circadian rhythms play a pivotal role in coordinating maternal–fetal signaling, trophoblast invasion, and nutrient exchange, all of which are critical for optimal pregnancy outcomes [18]. Altered circadian gene expression might therefore contribute to placental insufficiency, predisposing the fetus to adverse outcomes, such as intrauterine growth restriction. preterm birth, or vascular disorders like pre-eclampsia [40]. Alterations of CLOCK, Bmal1, PER1, and PER2 has been observed in the placental cells of pregnant women with vascular disorders like pre-eclampsia [41,42]. Based on our results, we propose that it is likely that the vascular stress associated with CVD perpetuates circadian dysregulation, compounding the pathological effects on placental function. For instance, the downregulation of CLOCK, Bmal1, PER1, and PER2 may be associated with increased hypoxia, oxidative damage, inflammation, and impaired angiogenic capacity [43], all of which have been previously observed in the placental tissue of women with CVD [9,44]. Future studies should be directed to explore the possible causes and potential consequences of circadian dysregulation in women affected by CVD.

On the other hand, we have demonstrated, for the first time, the epigenetic impact of suffering from CVD during pregnancy, as evidenced by an increased expression of HAT-1, and related molecules, which is indicative of histone modifications. Histone modifications, including methylation, acetylation, phosphorylation, and ubiquitination, are epigenetic processes that regulate gene expression by altering chromatin structure [45]. Histone modifications regulate key processes, like trophoblast differentiation and syncytiotrophoblast formation, essential for placental function. These modifications, such as acetylation (associated with activation) or deacetylation and certain methylation patterns (linked to inactivation), respond to environmental factors, like hypoxia, particularly during pregnancy [46]. The increased expression of HAT1 and the dysregulation of other epigenetic markers has been reported in the placental tissue of obese pregnant mice when compared to control pregnant mice [47], suggesting the relevance of the environment in the regulation of the epigenetic machinery. The pathogenic environment associated with CVD, including enhanced hypoxia, oxidative stress, or inflammation, could be potentially implicated in the aberrant expression of HAT-1 observed in our study. Despite that the role of HAT-1 has not yet been examined in the context of CVD, past works have evidenced the potential role of Histone deacetylases (HDACs)—implicated in histone deacetylation processes—in VVs, demonstrating an increase expression of HDAC-1, -2, -3, -5, and -7, triggering the development of CVD [48,49]. Further studies are warranted to explore and understand the possible role of HAT-1 and other HATs in the context of CVD, both in the venous and placental tissue.

HAT-1 is an enzyme mainly responsible for acetylating K5 and K12 on the N-terminus of H4 within newly formed H3/H4 dimers in the cytoplasm. In addition to this primary function, it also performs several other non-canonical roles, including transporting H3/H4 into the nucleus, regulating the expression of H4 genes, ensuring the stability of DNA replication forks, aiding in replication-coupled chromatin assembly, repairing DNA damage, silencing telomeres, affecting the epigenetic regulation of heterochromatin associated with the nuclear lamina, and supporting processes like acetylation and succinylation [23]. HAT-1 initially binds with RBBP7, forming a complex that leads to the acetylation of the H3/H4 heterodimer. This acetylated H3/H4 complex then interacts with the chaperone ASF-1, which helps transport the HAT1/RBBP7/H3/H4 complex from the cytoplasm to the nucleus [50,51,52]. The increased expression of H3/H4, RBBP7, and ASF-1 could be indicative of the possible role of HAT-1 and associated molecules in epigenetic regulation and other important processes in the placental tissue, although further studies are warranted to explore the possible mechanisms involved. In addition, it is important to note that histone acetylation is a complex and dynamic process regulated by multiple enzymes beyond HAT1, and other epigenetic modifications, such as DNA methylation, non-coding RNAs, and chromatin remodeling, also play crucial roles [20,53]. Since our study only assessed HAT1 expression without evaluating its enzymatic activity or direct histone acetylation levels, these findings should be interpreted with caution. Further investigations incorporating broader epigenetic markers, functional assays, and additional regulatory pathways are necessary to establish a more comprehensive understanding of epigenetic alterations in the placental tissue of women with CVD.

The increased expression of histones H3 and H4, along with rbbp7 and asf-1, in the placentas of women with CVD during pregnancy likely represents an adaptive response to the physiological and oxidative challenges posed by the disease. As core components of the nucleosome, histones H3 and H4 facilitate chromatin remodeling and enable epigenetic modifications, regulating critical genes involved in angiogenesis, nutrient transport, and oxidative stress responses [54,55]—processes augmented in the placenta of women with CVD [9]. This adaptive process also reflects an increased demand for DNA repair and chromatin stabilization, ensuring genomic integrity and possibly influencing fetal epigenetic programming and long-term development [9], although the specific implications for the fetus remain unclear.

RBBP7, like RBBP4, functions as a histone chaperone and plays an essential role in chromatin remodeling, cell cycle regulation, and DNA repair. These proteins interact with complexes, such as DREAM, MuvB, NuRD, and PRC2, modulating histone acetylation and methylation, supporting centromere function, and collaborating with BRCA1 to maintain genomic stability [56], Similarly, ASF-1, a histone H3–H4 chaperone, contributes to chromatin assembly during both replication-dependent and -independent processes and partners with other histone chaperones like CAF1 (chromatin assembly factor1) and HIRA (histone interacting protein A) [57]. Elevated levels of RBBP7 and ASF-1 in placental tissues may indicate a heightened need for dynamic chromatin remodeling to regulate gene expression, support increased cellular proliferation, and enhance DNA repair mechanisms in the placentas of women with CVD, although further studies are needed to confirm this hypothesis.

Finally, we also observed an increased expression of the anti-aging protein KLOTHO in the placentas of women with CVD when compared to the HC group. KLOTHO is a molecule implicated in multiple cellular processes and its dysregulation has been associated with accelerated aging and pathological events [58]. Despite being mainly produced in the kidneys, KLOTHO is also synthesized in other tissues including the human placenta [59]. Pregnant women present higher levels of circulating KLOTHO when compared to non-pregnant women, and the expression in the placenta of KLOTHO mRNA and protein varies according to fetal growth and increases with gestational age in physiological pregnancies [60,61]. However, the downregulation of this protein has been found in pathological pregnancies, both in the blood and placental tissue, which is indicative of accelerated placental aging [61,62,63,64]. Mechanistically, KLOTHO acts either as an obligate coreceptor for fibroblast growth factor 23 (FGF23) or as a soluble pleiotropic endocrine hormone (s-Klotho), inhibiting four pathways that have been linked to aging and aging-related processes in various ways: transforming growth factor β (TGF-β), insulin-like growth factor 1 (IGF-1), Wnt, and NF-κB [65]. The increased expression of IGF-1 and Wnt pathways—along with products activated by NF-κB, like the NLRP3 inflammasome, or by TGF-β, such as the PI3K/Akt/mTOR pathway—has been reported in the placentas of women with CVD [44,66]. KLOTHO downregulation can also promote a hypoxic environment through the overexpression of the hypoxia-inducible factor 1 (HIF-1) and alter glucose metabolism in the placental tissue [59], two major phenomena linked to the pathogenic environment of CVD [9,67]. Likewise, past works suggest that decreased KLOTHO expression in the placenta may favor oxidative stress and promote the development of calcification and apoptosis [61,68], which is also a footprint of CVD in the placental tissue during pregnancy [9]. The biological significance of the accelerated aging and decreased expression of KLOTHO in the placental tissue of women with CVD, however, should be deeply explored in future works.

Moreover, accelerated placental aging is also directly related to circadian dysregulation and epigenetic alterations, as they represent critical hallmarks of aging and health [69]. Future studies are warranted to connect all these entities, creating a molecular network explaining the pathological effects of CVD on the placental tissue.

## 5. Conclusions

**Figure 12:**
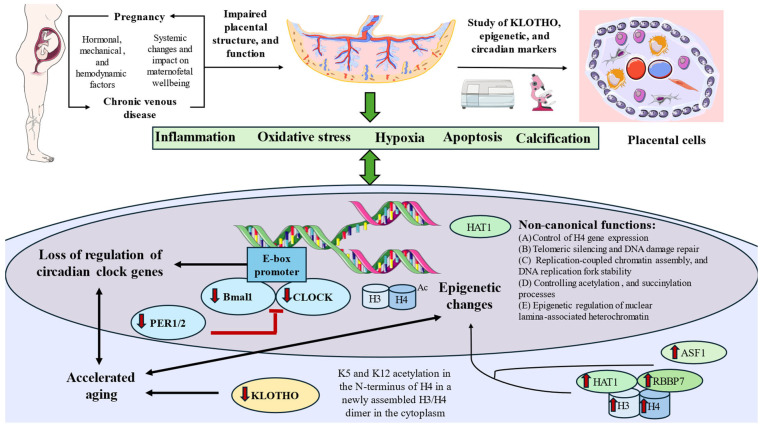
A summary of the main results obtained in this work and the potential consequences associated.

To our knowledge, we have evidenced for the first time that the placentas of pregnant females with CVD exhibit a significant dysregulation of circadian rhythms (noted by the reduced detection of core circadian regulators like Clock, Bmal1, per1, and per2), epigenetic changes (with increased levels of HAT-1 and related molecules like rbbp7, asf-1, H3, and H4), and accelerated aging, with a marked decrease in klotho expression. Figure 12 summarizes the main results obtained in this research. Overall, this study gains further insights into the pathogenic effects of CVD during pregnancy in maternofetal structures; however, more efforts are warranted in order to understand and extend the consequences of these changes.

## Figures and Tables

**Table 1:** The clinical characteristics of participants enrolled in the study. The same cohort was also studied in previous research.

Clinical Features	CVD (n = 98)	HC (n = 82)
Median age (IQR), years	33 (19–41)	34 (20–41)
Median gestational age (IQR), weeks	40 (39–41.5)	41 (39–42)
C-section delivery, n (%)	20 (20.4%)	15 (18.3%)
Vaginal delivery, n (%)	78 (79.6%)	67 (81.7%)
CVD CEAP 1, n (%)	59 (60.2%)	0
CVD CEAP 2, n (%)	32 (32.7%)	0
CVD CEAP 3, n (%)	7 (7.1%)	0
Prior pregnancies, n (%)	52 (53.1%)	31 (37.8%)
Prior abortions, n (%)	21 (21.4%)	14 (17.1%)
Regular periods of menstruation, n (%)	80 (81.6%)	66 (80.5%)
Sedentary profession, n (%)	65 (66.3%)	59 (71.9%)

**Table 2:** Primers for RT-qPCR techniques.

Gene	Sequence Fwd (5’→3’)	Sequence Rev (5’→3’)	Temperature
***TBP***	TGCACAGGAGCCAAGAGTGAA	CACATCACAGCTCCCCACCA	60 °C
***BMAL1***	AGGCCTGACTCACGTTTCCT	GAGGCTCATGATGACAGCCA	58 °C
***CLOCK***	AAAAGGAAACCCCGGAGAGC	CTCGCAGCATGTGACAACAG	60 °C
***PER1***	GCAGGCCAACCAGGAATACT	ACAGAAGCGGATAGGGGAGT	59 °C
***PER2***	ACTCCTCGGCTTGAAACGG	GCAGCCACTTGTAGATGGGT	60 °C
***HAT-1***	TCGGAAATGGCGGGATTTGG	TGTAGCCTACGGTCGCAAAG	57 °C
***H3***	GGGCCAACAGTTTCGGATTC	AAGCGCAGGTCGGTCTTAAA	60 °C
***H4***	GTGTGCTAAACGGGAGGGAA	CTTTCTGAGAGGGAGTGGGC	57 °C
***ASF-1A***	CAACCCGTTCCAGTTCGAGA	TCACTTTCTGCAGAGCCCAC	60 °C
***RBBP7***	GGAGGAGGCAGGAAAGAAGG	CCCCAGCACTAGCCAATGAA	59 °C
***KLOTHO***	TACAACAACGTCTTCCGCGA	TGCTCTCGGGATAGTCACCA	59 °C

**Table 3:** Antibodies for immunohistochemistry assays.

Antigen	Species	Dilution	Provider	Protocol Specifications
**Bmal1**	Rabbit monoclonal	1:1000	Abcam (ab230822)	------
**Clock**	Rabbit polyclonal	1:100	Abcam (ab3517)	------
**Per1**	Rabbit polyclonal	1:20	Abcam (ab254751)	------
**Per2**	Rabbit polyclonal	1:100	Abcam (ab200388)	------
**Hat-1**	Rabbit monoclonal	1:1000	Abcam (ab193097)	------
**H3**	Rabbit polyclonal	1:100	Abcam (ab1791)	------
**H4**	Rabbit monoclonal	1:500	Abcam (ab51997)	------
**Asf-1**	Rabbit polyclonal	1:100	Abcam (ab235358)	------
**Rbbp7**	Rabbit monoclonal	1:2000	Abcam (ab259957)	------
**Klotho**	Rabbit monoclonal	1:100	Abcam (ab181373)	------
**IgG**	Rabbit-Mouse monoclonal	1:1000	Sigma-Aldrich (RG96/B5283)	-----

## Data Availability

The data used to support the findings of the present study are available from the corresponding author upon request.
